# Decontamination of mycoplasma-contaminated *Orientia tsutsugamushi* strains by repeating passages through cell cultures with antibiotics

**DOI:** 10.1186/1471-2180-13-32

**Published:** 2013-02-08

**Authors:** Motohiko Ogawa, Tsuneo Uchiyama, Masaaki Satoh, Shuji Ando

**Affiliations:** 1 Department of Virology 1, National Institute of Infectious Diseases/1-23-1, Toyama, Shinjuku-ku, Tokyo, 162-8640, Japan; 2 Institute of Health Biosciences, The University of Tokushima Graduate School/3-18-15, Kuramoto-cho, Tokushima, 770-8503, Japan

**Keywords:** *Orientia tsutsugamushi*, Intracellular bacteria, Mycoplasma, Contamination, Elimination, Cell culture, Antibiotics

## Abstract

**Background:**

Mycoplasmas-contamination of *Orientia tsutsugamushi*, one of the obligated intracellular bacteria, is a very serious problem in *in vitro* studies using cell cultures because mycoplasmas have significant influence on the results of scientific studies. Only a recommended decontamination method is to passage the contaminated *O. tsutsugamushi* strains through mice to eliminate only mycoplasmas under influence of their immunity. However, this method sometimes does not work especially for low virulent strains of *O. tsutsugamushi* which are difficult to propagate in mice. In this study, we tried to eliminate mycoplasmas contaminants from both high virulent and low virulent strains of the contaminated *O. tsutsugamushi* by repeating passage through cell cultures with antibiotics *in vitro*.

**Results:**

We cultured a contaminated, high virulent strain of *O. tsutsugamushi* using a mouse lung fibroblasts cell line, L-929 cell in the culture medium containing lincomycin at various concentrations and repeated passages about every seven days. At the passage 5 only with 10 μg/ml of lincomycin, we did not detect mycoplasmas by two PCR based methods whereas *O. tsutsugamushi* continued good growth. During following four passages without lincomycin, mycoplasmas did not recover. These results suggested that mycoplasmas were completely eliminated from the high virulent strain of *O. tsutsugamushi*. Furthermore, by the same procedures with 10 μg/ml of lincomycin, we also eliminated mycoplasmas from a contaminated, low virulent strain of *O. tsutsugamushi*. Our additional assay showed that 50 μg/ml of lyncomycin did not inhibit the growth of *O. tsutsugamushi*, although MICs of many mycoplasmas contaminants were less than 6 μg/ml as shown previously.

**Conclusion:**

Our results showed an alternative method to eliminate mycoplasmas from the contaminated *O. tsutsugamushi* strains in place of *in vivo* passage through mice. Especially this notable method works for the decontamination not only from the high virulent strain also from the low virulent strain of *O. tsutsugamushi*. For further elimination, lincomycin at the limit concentration, which does not inhibit the growth of *O. tsutsugamushi*, can possibly eliminate most mycoplasmas from contaminated *O. tsutsugamushi* strains.

## Background

The contamination of cell cultures by mycoplasmas is a serious problem because these bacteria have multiple effects on cell cultures and also have a significant influence on the results of scientific studies. The mycoplasmas are not harmless bystanders and thus cannot be ignored in the cell cultures.

Various elimination methods were previously reported [1-3]. These methods are mainly based on four general procedures, physical, chemical, immunological and chemotherapeutic treatment. The physical procedures include heat treatment and filtration. The chemical procedures, treatments to detergents and other chemicals which are effective only against mycoplasmas, but not against host cells. The immunological procedures include *in vitro* co-culture with macrophages and specific anti-mycoplasmas antisera and *in vivo* passage thorough mice. The chemotherapeutic procedures are mainly antibiotics treatments that are kills mycoplasmas.

*Orientia tsutsugamushi*, which is the causative agents of scrub typhus is one of the obligated intracellular bacteria [4]. The mycoplasmas-contaminations of *O. tsutsugamushi* is also very serious in the *in vitro* studies using cell cultures. Furthermore the most effective methods for elimination of mycoplasmas can not be applied for decontamination of *O. tsutsugamushi* strains because these methods also inhibit the growth of *O. tsutsugamushi*. Decontamination methods should have strong effect on mycoplasmas, but have minimum or no effect on *O. tsutsugamushi*. Only the recommended decontamination method is to passage the contaminated *O. tsutsugamushi* strains through mice. Mouse immunity possibly eliminates only mycoplasmas, although *O. tsutsugamushi* can survive in its target cells, mainly endothelial cells, splenocytes and hepatocytes. In fact, homogenized spleen of infected mice is generally used for the next inoculation. However, this method sometimes does not work especially for low virulent strains of *O. tsutsugamushi* because they are generally difficult to propagate in mice.

Some of the antibiotics, which are used for elimination of mycoplasmas from tissue culture, are supposed to have less effect against *O. tsutsugamushi* according to the differences of minimum inhibitory concentrations (MICs) of antibiotics between mycoplasmas [5-7] and *O. tsutsugamushi*[8]. In this study, we tried to eliminate mycoplasmas from contaminated *O. tsutsugamushi* strains by repeating passages through cell cultures with antibiotics *in vitro*.

## Results and discussion

According to the MICs of antibiotics in the previous reports [5,7-9], we used two antibiotics, lincomycin and ciprofloxacin for elimination of mycoplasmas from the contaminated *O.tsutsugamushi* strains (Table 1). Both lincomycin and ciprofloxacin are effective against mycoplasmas. Unfortunately there is no available information about the MICs of lincomycin against *O. tsutsugamushi.* However, according to the MICs of lincomycin against gram-negative bacteria [10], lincomycin is supposed to be much less effective against *O. tsutsugamushi* because *O. tsutsugamushi* is one of the gram-negative bacteria. For the example, the MICs of lincomycin against *Escherichia coli*, one of the typical gram gram-negative bacteria are more than 50 times higher than those against mycoplasmas. Ciprofloxacin was also less effective against *O. tsutsugamushi*. The MICs of ciprofloxacin against *O. tsutsugamushi* are about 3 to 200 times higher than those against mycoplasmas (Table 1).

**Table 1:** Minimum inhibitory concentrations (MICs) of antibiotics used in this study

**Antibiotics**	**Drug class**	**MICs against*Orientia***^**a)**^	**MICs against mycoplasmas**^**b)**^
Lincomycin	Lincosamide	No available data	**0.25–2 μg/mL**
Ciprofloxacin	New Quinolone	**6.25–25 μg/mL**	**0.125–2 μg/ml**
Gentamicin	Aminoglycoside	No available data^c)^	2.5–500 μg/mL
Kanamicin	Aminoglycoside	No available data	2.5–500 μg/mL
Minocycline	Tetracycline	0.024–0.195 μg/mL	0.016–32 μg/mL

MICs were obtained from previous reports. a) from [8] and b) from [5-7].

c) Gentamycin was not effective against *Orinetia tsutsugamushi* in a mouse model [25].

Our result of the direct sequencing showed that Ikeda and Kuroki strains of *O. tsutsugamushi* were contaminated with *Mycoplasma hominis* and *M. orale* respectively. *M. hominis* and *M. orale* are 10 to 30% of contaminants of cell cultures (Table 2) [11]. Previous reports showed that *M. fermentas, M. hyorhinis, M. arginini* and *Acholeplasma laidlawii* are the most common contaminants as well as *M. hominis* and *M. orale.* More than 90% of the contaminants were caused by these six mycoplasmas [11,12]. The TaqMan PCR and the nested PCR can detect not only all the 6 most common contaminants also some other mycoplasmas. These facts suggested that the detection methods were very reliable to monitor mycoplasmas-contaminations in this study.

**Table 2:** Major mycoplasmas, and their detection and sequencing methods in this study

**Species**		**PCR for detection**		**PCR for Sequencing**^**d)**^		
	**Frequency of contamination**^**a)**^	***tuf*gene (TaqMan PCR)**^**b)**^	**16S-23S ribosomal RNA intergenic region (nested PCR)**^**c)**^	**Match of new PCR primers**	**Strains**	**Sequence ID**
Most common contaminant species						
*Mycoplasma fermentans*	10%-20%	+	+	Match	human B cell lymphoma contaminants, 16054780	AY838558
*Mycoplasma hyorhinis*	10%-40%	+	+	Match	HUB-1	NC_014448.1
*Mycoplasma orale*	20%-30%	+	+	Partial Match	ATCC 23714D	gi|315440428
*Mycoplasma arginini*	20%-30%	No Data	+	Partial Match	G230	gi|290575476
*Acholeplasma laidlawii*	5%-20%	+	+	Match	PG-8A	CP000896
*Mycoplasma hominis*	10%-20%	+	+	Match	ATCC 23114	M57675
Other species						
*Mycoplasma arthritidis*	No Data	+	No Data	Match	158L3-1	NC_011025.1
*Mycoplasma bovis*	No Data	+	No Data	Match	PG45	NC_014760.1
*Mycoplasma buccale*	No Data	+	No Data	No data	-	-
*Mycoplasma faucium*	No Data	+	No Data	No data	-	-
*Mycoplasma gallisepticum*	No Data	+	No Data	Match	PG31	X16462
*Mycoplasma genitalium*	No Data	+	+	Match	ATCC33530	X16463
*Mycoplasma hyopneumoniae*	No Data	+	No Data	Match	7448	NC_007332.1
*Mycoplasma penetrans*	No Data	+	No Data	Match	HF-2	NC_004432.1
*Mycoplasma pneumoniae*	No Data	+	+	Match	FH	X55768
*Mycoplasma primatum*	No Data	+	No Data	No data	-	-
*Mycoplasma salivarium*	No Data	+	+	Partial Match	ATCC 23064D	gi|313575713
*Ureaplasma parvum*	No Data	+	No Data	Match	ATCC 33697	AF270770
*Mycoplasma zalophi*	No Data	No Data	No Data	Match	CSL 4296	gi|148536300
*Mycoplasma mycoides*	No Data	No Data	No Data	Match	PG1	gi|126252003
*Mycoplasma capricolum*	No Data	No Data	No Data	Match	ATCC 27343	gi|83319253
*Mycoplasma agalactiae*	No Data	No Data	No Data	Match	PG2	gi|148291314
*Mycoplasma pyrum*	No Data	No Data	+	No data	-	-

a) Upper 6 species of mycoplasmas are the most common contaminants of cell cultures [11,12].

b) This broad-range TaqMan PCR can detect many species of mycoplasmas [22].

c) This nested PCR is highly sensitive, and it is used to check for mycoplasma contamination in the Cell Bank of BioResource Centre, Riken Tsukuba Institute, Tsukuba, Ibaraki, Japan [21].

d) PCR assay for sequencing of mycoplasmas designed in this study. Partial Match means that 2 or 3 of the total of 4 nested-PCR primers match to available regions of the *tuf* gene on the public database.

**Figure 1:**
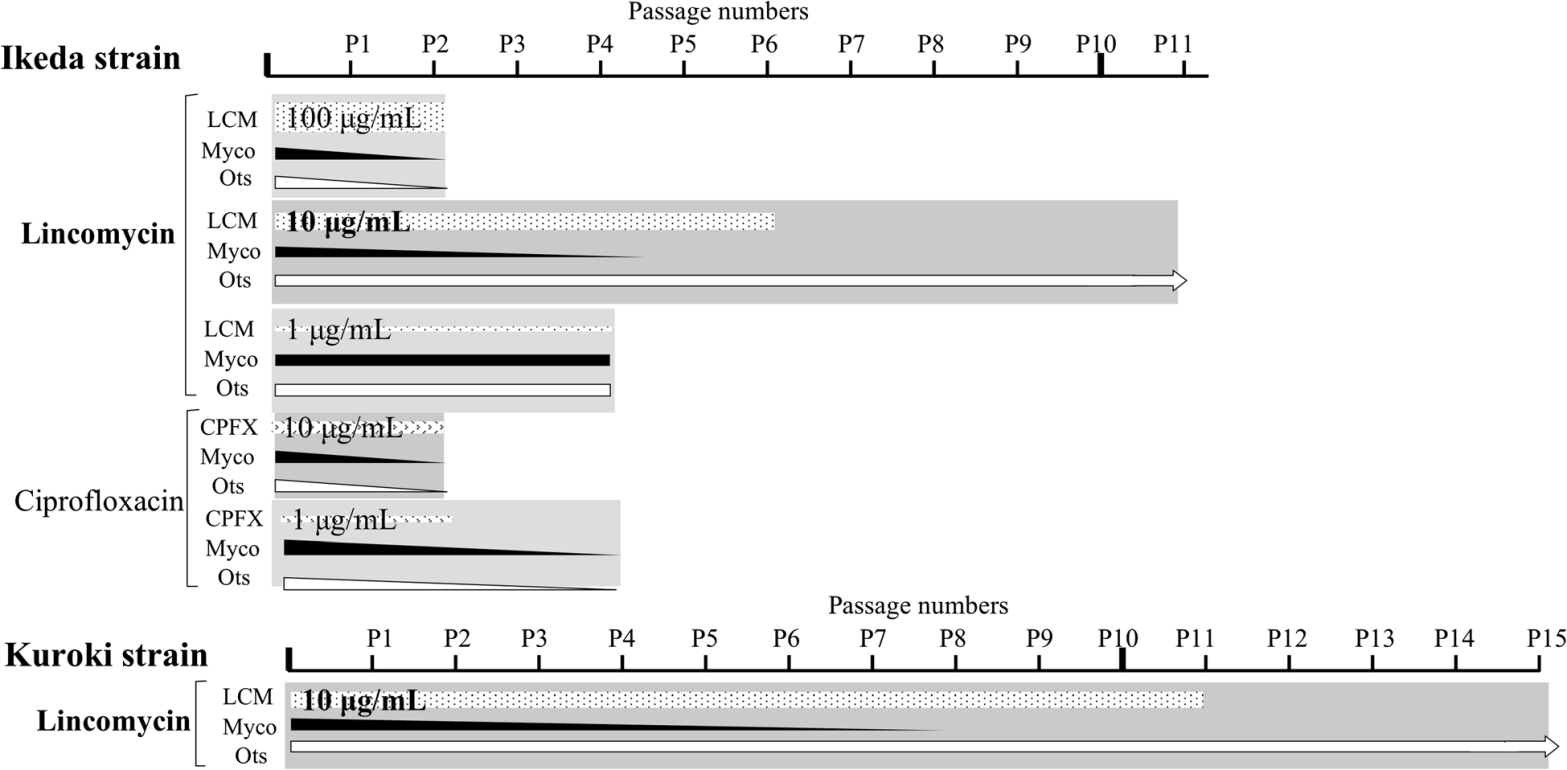
**Illustrations of decontamination of mycoplasma-contaminated *O. tsutsugamushi *strains by repeating passage through cell cultures with antibiotics. ** Ikeda is a high virulent strain, whereas Kuroki is a low virulent strain, which is difficult to propagate in mice. LCM: lincomycin, CPFX: ciprofloxacin, Myco: mycoplasmas, Ots: *O. tsutsugamushi.*

For elimination of mycoplasmas, we first cultured a contaminated, high virulent Ikeda strain of *O. tsutsugamushi* using L-929 cell in the culture medium containing lincomycin and ciprofloxacin and repeated the passages (Figure 1). Lincomycin and ciprofloxacin were used at 100, 10 and 1 μg/ml. However, ciprofloxacin at 100 μg/ml were cytotoxic against L-929 cell in the first assay and was omitted from the further analyses. We checked mycoplasma-contaminations and *O. tsutsugamushi-growth* at each passage by the two PCR based methods and/or an immunofluorescent (IF) staining (see Additional file 1). From the passage 1 to 2 with 10 μg/ml of lincomycin, the real-time PCR showed that mycoplasmas decreased, whereas *O. tsutsugamushi* did not decrease. At the passage 4 with the same concentration of lincomycin, the real-time PCR did not detect mycoplasmas, however the nested PCR still detected them. At the passage 5, both the real-time PCR and the nested PCR did not detect mycoplasmas, whereas the flourish growth of *O. tsutsugamushi* was observed by IF staining. We continued to culture with lincomycin until the passage 6. During following passages from 7 to 10 without lincomycin, mycoplasmas did not recover. These results clearly showed that mycoplasmas were completely eliminated from *O. tsutsugamushi*-infected cells. However, the cultivation with 100 μg/ml of lincomycin as well as 10 and 1 μg/ml of ciprofloxacin decreased both mycoplasmas and *O. tsutsugamushi-*growths, whereas the cultivation with 1 μg/ml of lincomycin did not influence the neither growths.

By the same procedure of Ikeda strain, we cultured a contaminated, low virulent Kuroki strain of *O. tsutsugamushi* with lincomycin at 10 μg/ml (Figure 1). Mycoplasmas and *O. tsutsugamushi* were monitored by the nested PCR and the IF assay respectively (see Additional file 2). At the passage 8, the nested PCR did not detect mycoplasmas. We then continued to cultivate it with lincomycin until the passage 11. During following passages from 12 to 14 without lincomycin, mycoplasmas did not recover. These results showed that we successfully eliminated mycoplasmas also from the low virulent Kuroki strain. The elimination length of Kuroki strain was longer than that of Ikeda strain probably because numbers and/or antibiotics-susceptibility of the contaminated mycoplasmas were different. For further elimination of mycoplasmas from other strains of *O. tsutsugamushi*, we should first evaluate a maximum concentration of lincomycin that does not influence *O. tsutsugamushi-*growth, and then apply it for decontamination because maximum effects against mycoplasmas are necessary to eliminate them for a short time and to avoid producing lincomycin-resistant mycoplasmas [13-15] during repeating passages. Our additional assay showed that lincomycin at 25 μg/ml did not affect the growth (the virulent strain), whereas 50 μg/ml slightly decreased (did not inhibit) the growth in the IF assay (Table 3). Many previous reports about antibiotics-susceptibilities of isolated mycoplasmas showed that MICs of lyncomycin against *M. hominis*, *M. fermentas* and *A. laidlawii*, which are the major contaminants, were less than 6 μg/ml (0.025 to 6 μg/ml) [5,16-18]. In actual, a previous report showed that lincomycin at 50 μg/ml successfully eliminated the other major contaminants of mycoplasmas, *M. hyorhinis* and *M. hominis* from cell cultures [19]. However, a previous report showed that some isolates of *M. hyorhinis* were highly resistant to lyncomycin (MICs > 100 μg/ml) [14] and a few reports showed that other species of mycoplasmas but not major species of contaminants were highly resistant to lyncomycin [13,15]. Considering these facts, lincomycin at 50 μg/ml can possibly eliminate the contaminants from many of other contaminated strains of *O. tsutsugamushi*, although it might not be effective for all the cases.

**Table 3:** **The growth of *O. tsutsugamushi *at the various concentrations of lincomycin**

	**Concentrations of lincomycin in the culture medium**			
	12.5 μg/ml	25 μg/ml	50 μg/ml	100 μg/ml
*O. tsutsugamsuhi*-growth^a)^	+++	+++	++	-

a) A virulent Ikeda strain was cultivated using L-929 cell in the culture medium containing lyncomycin at the indicated concentrations. The growth was observed by the immunofluorescent staining.

## Conclusions

Our results showed an alternative method to eliminate mycoplasmas from the mycoplasma-contaminated strains of *O. tsutsugamushi* in place of *in vivo* passage through mice. Especially this new method works for the decontamination not only from the high virulent strain also from the low virulent strain of *O. tsutsugamushi, which* is difficult to propagate in mice. For further elimination, lincomycin at the limit concentration, which does not inhibit the growth of *O. tsutsugamushi*, can possibly eliminate most mycoplasmas from contaminated *O. tsutsugamushi* strains.

## Methods

### Cell lines

A mycoplasmas-free L-929 cell (a mouse fibroblast cell line, JCRB9003) [20] was grown in Eagle’s minimum essential medium (MEM, Wako Co. Ltd., Tokyo, Japan) supplemented with 5 to 10% of mycoplasma-free, heat-inactivated FCS (Sigma-Aldrich Japan Co. LCC., Tokyo, Japan) at 37°C in 5% CO_2_.

### Mycoplasmas-contaminated *O. tsutsugamushi* strains for elimination

A mycoplasmas-contaminated high virulent Ikeda strain and a low virulent Kuroki strain of *O. tsutsugamushi* were used for elimination. These strains were accidentally contaminated during a long passage history probably because mycoplasmas-contaminated cell culture was used for propagation of these strains. The mycoplasma-free L-929 cell was used for propagation as mentioned in the previous section.

### Detection and quantification of mycoplasmas

Major mycoplasmas are listed in Table 2. Upper 6 species are the most common contaminants in cell cultures [11,12]. In order to monitor mycoplasmas, we extracted DNA from *O. tsutsugamushi*-infected L-929 cell with a commercial DNA extract kit (Tissue genomic DNA extraction mini kit, Favorgen biotech corporation, Ping-Tung, Taiwan) and detected mycoplasmas by two high sensitive and broad range PCR based methods for detection, the nested PCR [21] and the real-time PCR (TaqMan PCR) [22]. The nested PCR is used to check mycoplasma*-*contaminations in the Cell Bank of Bioresource Centre, Riken Tsukuba institute, Tsukuba, Ibaraki, Japan. For determination of mycoplasma species, we designed new sequencing primers against *tuf* gene (Table 2). These designed primers matched *tuf* gene of 19 mycoplasmas on the public database. All the primers and the probe are listed in Table 4.

**Table 4:** Primers and probes for detection and sequencing in this study

**Targets**	**Assay**	**Name**	**Primers and probes**
Mycoplasmas			
*tuf* gene^a)^	real-time PCR	Mollicutes 414F	5'-TCCAGGWCAYGCTGACTA-3'
		Mollicutes 541R	5'-ATTTTWGGAACKCCWACTTG-3'
		Probe 451F^a)^	5'-GGTGCTGCACAAATGGATGG-3'
*tuf* gene	Sequencing 1st	Myco-tuf-F1	5'-HATHGGCCAYRTTGAYCAYGGKAAAA-3'
		Myco-tuf-F2	5'-ATGATYACHGGDGCWGCHCAAATGGA-3'
	Sequencing 2nd	Myco-tuf-R1	5'-CCRCCTTCRCGRATDGAGAAYTT-3'
		Myco-tuf-R2	5'-TKTRTGACGDCCACCTTCYTC-3'
16s-23s rRNA intergenic spacer region	nested PCR 1st	MCGpF11	5'-ACACCATGGGAGYTGGTAAT-3'
		R23-1R	5'-CTCCTAGTGCCAAGSCATYC-3'
	nested PCR 2nd	R16-2	5'-GTGSGGMTGGATCACCTCCT-3'
		MCGpR21	5'-GCATCCACCAWAWACYCTT-3'
*Orientia tsutsugamushi*			
47kDa common antigen coding gene	real-time PCR	Ots-47k-F	5'-AATTCGTCGTGGTATGTTAAATG-3'
		Ots-47k-R	5'-AGCAATTCCACATTGTGCTG-3'
		Ots-47k-P ^b)^	5'-TGCTTAATGAATTAACTCCAGAATT-3'

a) Locked nucleic acid (LNA) bases (underlined) and was synthesized with the fluorescent reporter 6-carboxyfluorescein (FAM) covalently coupled to the 5’ end and a dark quencher to the 3’ end.

b) TaqMan probe was synthesized with the fluorescent reporter 6-carboxyfluorescein (FAM) covalently coupled to the 5’ end and a dark quencher to the 3’ end.

### Detection of *O. tsutsugamushi*

To monitor the growth of *O. tsutsugamushi*, we used a real-time PCR against the gene encoding 47kDa common antigen (Table 4). We extracted DNA from *O. tsutsugamushi*-infected L-929 cell as mentioned in the previous section and performed the real-time PCR according to the general procedure [23]. We also used an IF staining to monitor the growth of *O. tsutsugamushi*. In this staining, human convalescent sera of a scrub typhus patient, which were permitted by the ethics committee (number 255), and anti-human antibody conjugated with AlexaFluor®488 (Life technologies Japan Ltd, Tokyo, Japan) were used. A part of the infected cells were harvested and fixed on a glass slide with ice cold acetone and then the slide was applied for the IF staining according to the previous reports [24].

### Antibiotics

Lincomycin (Wako Pure Chemical Industries, Ltd., Osaka, Japan) and ciprofloxacin (Wako Pure Chemical Industries, Ltd., Osaka, Japan) were used for elimination of mycoplasmas in this study. Kanamycin and gentamycin are routinely used for propagation of *O. tsutsugamushi* to avoid accidental bacterial contamination in our laboratory because they do not influence *O. tsutsugamushi*-growth [25].

### Elimination of mycoplasmas from *O. tsutsugamushi*-infected cells with antibiotics

We cultured the contaminated strains of *O. tsutsugamushi* using L-929 cell in the culture medium containing lincomycin and ciprofloxacin at 100, 10 and 1 μg/ml in 25cm2 tissue culture flask, and repeated passages about every seven days. At each passage, the infected cells were harvested. One-third of the harvested cells was used for the next inoculation, another one-third was used for DNA extraction, and the remaining one-third was frozen and stocked. Elimination of mycoplasmas was checked by the nested PCR and/or real-time PCR. The growth of *O. tsutsugamushi* was monitored by the real-time PCR and/or the IF staining.

## Competing interests

All authors declare that they have no competing interest.

## Authors’ contribution

MO carried out the entire part of this study. TU carried out DNA sequences and some genetic analyses of mycoplasmas. MS and SA helped the passages of *O. tsutsugamushi* in cell culture with lyncomycin and checked mycoplasmas and *O.tsutsugamushi* by PCR and IF assay. All authors read and approved the final manuscript.

## Acknowledgements

This study was financially supported by a grant from the Ministry of Health, Labour and Welfare, Japan (number H21-Shinkou-Ippan-006 and H23-Shinkou-Ippan-007 from 2010 to 2012).

## Supplementary Material

Additional file 1 **Decontamination of a mycoplasma-contaminated, high-virulent strain of *Orientia tsutsugamushi *(Ikeda strain) by repeated passages with antibiotics.** Click here for file

Additional file 2 **Decontamination of a mycoplasma-contaminated, low-virulent strain of *Orientia tsutsugamushi *(Kuroki strain).** Click here for file
